# Investigating Additive Effects on α-Glycine Growth through the Measurement of Facet Specific Growth Rates

**DOI:** 10.1021/acs.cgd.5c00028

**Published:** 2025-02-13

**Authors:** Caroline Offiler, Roger J. Davey, Aurora J. Cruz-Cabeza, Thomas Vetter

**Affiliations:** † Department of Chemical Engineering, University of Manchester, Manchester M13 9PL, United Kingdom; ‡ Department of Chemistry, Durham University, Durham DH1 3LE, United Kingdom; § H. Lundbeck A/S, Valby 2500, Denmark

## Abstract

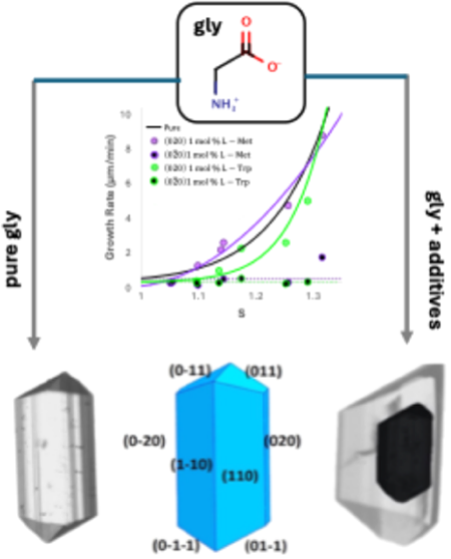

The presence of trace amounts of additives during crystal growth can have a significant impact on the physical properties of the crystallizing substrate (e.g., crystal morphology, purity, polymorphic phase, or growth kinetics). In this work, we report the growth of α-glycine crystals (α-gly) in the presence of a variety of diverse additives: two l-amino acids, two organic acids, α-iminodiacetic acid, and two chloride salts. Growth rate data from imaging, together with analytical techniques such as X-ray photoelectron spectroscopy (XPS) and fluorescence microscopy, are used to observe which facet growth is impacted by the additive and to what extent. Relating these findings to the α-gly crystal structure provides explanations for the observed effects. Specifically, the growth inhibition of the (02̅0) facet α-gly in the presence of l-tryptophan and l-methionine shows how the prochirality of glycine results in two symmetrically equivalent facets growing at different rates. In the presence of malonic acid and salicylic acid, growth of the {011} facets is inhibited as a result of the interaction of deprotonated acids at the {011} surfaces. We find α-iminodiacetic acid to be an extremely effective inhibitor of α-gly, stopping the growth of both the {011} and {020} facets. We correlate the effectiveness of α-iminodiacetic acid to its structural similarity to gly, allowing it to easily block the growth of two α-gly facets. Finally, we observe the incorporation of the metal ions Fe(II), Cu(II), and Zn(II) into the {011} facets of α-gly. Interestingly, in the cases of Cu(II) and Zn(II), the incorporation of the metals into the α-gly lattice does not cause a noticeable change in the growth rates. The formation of coordination complexes containing the metal ions and glycine ligands allows for the observed incorporation of the metals into the α-gly lattice with limited disturbance to its crystal growth.

## Abstract 2

Growth rate data of α-glycine facets in the presence of a varied selection of additives is presented together with a molecular-level rationalization of the mechanisms.

## 1 Introduction

Impurities are very difficult to avoid during crystal growth; they are often found in starting materials, as byproducts, or unreacted species present in the crystallization environment. In some cases, impurities may be added on purpose (additives) with the sole purpose of tuning crystal morphologies to a more desirable shape^1−4^ or blocking the growth of a specific polymorph.^5^ Such additives (even at relatively low levels) can have a significant impact on crystal growth kinetics, affecting the various crystal facets to a different extent and leading to changes in crystal morphologies.^6^ Since crystal morphology is important in downstream processes during product development^7^ and impacts materials properties such as dissolution rate or bioavailability,^8^ understanding and controlling growth additives to tailor crystal morphologies is a powerful tool for materials development. The first step in designing a morphology modifying additive^9^ is to explore the crystal morphology of the material in question and decide which facets need to be modified in order to achieve the desired morphological outcome. Through a crystal morphology engineering approach^8,9^ and with the aid of molecular simulations,^6^ it is then possible to anticipate which molecular functionality and stereochemistry an additive needs in order to interact with the desired facets and hence impact its growth.^10,11^

Glycine (gly) is the simplest amino acid of fundamental importance to life and has a wide variety of commercial uses, including as a food and drug additive (due to its sweet flavor and action as an antioxidant),^12^ as a reagent in herbicide production, and as a dietary supplement, with some recent research suggesting it can improve sleep.^13^ As of 2010, gly is present in 174 pharmaceutical drugs as an emulsifier, sweetener, or solubilizing agent.^14^ In addition, gly is a compound with great historical importance when it comes to the study and understanding of crystallography and crystal growth, driving many of the seminal studies carried out by the Weizmann group between 1983 and 2003.^9−11,15^ Gly is the first amino acid to be reported as polymorphic;^16,17^ it has been used as a model compound to demonstrate direct assignment of absolute configuration,^18^ to design tailor-made additives for both morphological and polymorph control,^19^ and to gain insights into solvent effects on polymorphism, including the idea of a relay mechanism;^15^ Langmuir monolayers of gly have been used to induce ice nucleation;^20,21^ gly polymorphs have been purposely nucleated and grown with strong lasers,^22,23^ electric^24^ and magnetic fields;^25^ and in the presence of numerous additives (some accelerating its growth)^26^ and surfactants,^27^ from emulsions and at different pHs^28^ leading to different polymorphs;^29^ gly has also been shown to have pyro-,^30^ piezo-^31^ and ferroelectric^32^ properties; and the list goes on. In Boldyreva’s words, gly is a “gift that keeps on giving.”^33^

**Figure 1:**
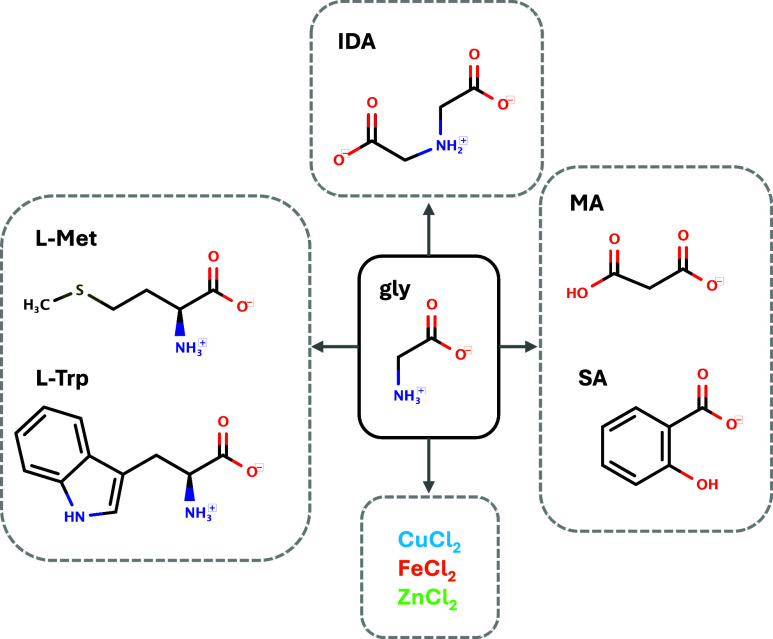
Molecular structure of the additives studied in this work. The dominant species under the conditions of crystallization are depicted.

In the present work, we study the impact of various additives (Figure 1) on the facet specific growth rates of α-gly in water.^34^ For this, we use a crystal growth cell operated under flow conditions (FC) with recorded images of growing crystals analyzed with a novel image analysis method that returns facet specific crystal growth rates.^34,35^ While flow conditions can introduce various complex hydrodynamic effects such as variation of growth rates as a function of the crystal orientation relative to the flow direction (e.g., form I benzamide),^35^ we have previously shown that α-gly is not affected by this since α-gly growth rates under flow and stagnant conditions are identical.^34^ We have chosen a varied set of additives which can be categorized into four different groups (Figure 1): (a) two chiral l-amino acids, (b) iminodiacetic acid (IDA), (c) two organic carboxylic acids of different structure, and (d) three metal chloride salts. For the chiral l-amino acids (S handedness), two compounds with different substituents on the α carbon were chosen: l-methionine (l-Met) with a long chain substituent and l-tryptophan (l-Trp) with a bulkier substituent containing a terminal indole ring. For the organic acids, we have chosen malonic acid (MA) and salicylic acid (SA). For the chloride salts, we have chosen CuCl_2_, FeCl_2_, and ZnCl_2_.

In terms of speciation in solution under the used crystallization conditions (water pH ∼ 5), l-Met and l-Trp exist as neutral zwitterions while IDA (p*K*_a1_ = 2.12, p*K*_a2_ = 2.90, p*K*_a_[base] = 9.63), MA (p*K*_a1_ = 2.83 and p*K*_a2_ = 5.69), and SA (p*K*_a1_ = 2.98) exist as monovalent anions, and the salts exist as dissociated ion pairs. Consequently, all a–c categories of additives would have the ability to attach to α-gly growing faces with either the carboxylate moiety or the −NH_3_^+^ moiety common with gly or complexate gly in the case of the metal ions (Figure 1).

## 2 Experimental Section

### 2.1 Materials

α-Glycine (≥99%), l-methionine (≥98%), l-tryptophan (≥98%), iminodiacetic acid (98%), salicylic acid (≥99%), and copper(II) chloride (99%) were purchased from Sigma-Aldrich. A 1.0 M hydrochloric acid solution was purchased from Fluka; malonic acid (99%) and zinc(II) chloride (99.95%) were purchased from Alfa Aesar, and iron(II) chloride tetrahydrate (99+%) was purchased from Arco Organics. All chemicals were used without further purification. Ultrapure type one water obtained from a Direct Q 3 UV purifier was used throughout.

### 2.2 Solubility and Definition of Supersaturation Ratio

The solubility of pure α-gly was determined previously.^7^ For each additive studied, the α-gly aqueous solubility at 15 °C in the presence of 1 mol % of the additive was measured using the gravimetric method. For this, excess gly, together with 1 mol % of the additive, was stirred (magnetic flee) in water for 24 h at 15 °C. Because gly and the additives used here are zwitterionic or salts with high solubility in water, the equilibrium is reached fast and 24 h of stirring is enough to reproduce the experimental solubility of gly with accuracy.^7^ Three 2.5 mL samples of saturated solution were then extracted and filtered (using a 0.45 μm syringe filter), and their gly content was determined gravimetrically. The average solubility (in mole fraction) and the standard deviation were calculated. Knowledge of the solubilities allowed for the calculation of supersaturation ratio (*S*, eq 1), where *S* is defined as the ratio between the mole fraction of gly in the solution (*x*_gly_) and the solubility for α-gly in water in the presence of the appropriate additive content (*x*_α-gly_^eq^). We note that our calculation of supersaturation neglects the impact of activity coefficients and assumes pure solid phases.
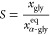
1

### 2.3 Seed Crystals

α-Gly seed crystals were prepared by slow evaporation from water. Aqueous solutions of gly were prepared by dissolving approximately 0.2 g of gly per gram of water at 50 °C; they were then cooled to room temperature and left to evaporate over time until crystals of appropriate sizes (∼700 μm) were obtained. Crystals were then isolated with the aid of vacuum filtration and analyzed under a polarized microscope. An Axioplan 2 Microscope (Zeiss, Jena) was used to examine the quality of the seed crystals under cross polarizers in order to select good quality single crystals for the experiments (discarding crystals displaying twinning, agglomeration, or visible defects).

### 2.4 Measurements of Experimental Crystal Growth Rates

Growth rates of the α-gly seed crystals were measured in the crystal growth cell under a constant flow of solution, our recently developed setup, which has been described in detail elsewhere.^34^ Briefly, seed crystals are placed inside a quartz flow cell, which is placed within a water bath, built with clear Perspex windows, and held at 15 °C. An light-emitting diode (LED) backlight is positioned below the water bath, and a camera is positioned above. Initially, the seed crystals are dissolved by flowing an undersaturated solution, and then, the feed solution changed to that of a supersaturated solution of the desired concentration. Cycles of dissolution and growth are repeated to explore a full range of supersaturations. The camera captures images of the growing crystals, with the acquisition rate set to one image every 10 s. For each additive, aqueous solutions of gly with 1 mol % of additive were prepared at various supersaturation values. For each experiment, the solution is pumped at a rate of 35 g/h from a solution reservoir through the flow cell (0.8 mL in volume) (FC), making use of a gear pump connected to a flow meter. Each experiment lasts approximately 1 h. The images of our growing crystals are then analyzed with the aid of a self-made MATLAB code, described in detail in our previous work.^34^ The fixed centroid version of the code was used to allow any unsymmetrical growth to be seen; this is important to this work as chiral amino acids are known to cause unsymmetrical growth in α-glycine.^10^ For symmetrically growing facets, the rate from the centroid to the related facets is calculated, and an average of the rate is given with its standard deviation. For facets whose growth symmetry is disrupted by chiral additives, a single rate is derived per unique facet and growth condition with no standard deviation.

### 2.5 Elemental Analysis

Large (>0.5 cm) α-gly crystals grown at 15 °C from aqueous solutions (*S* = 1.45) in the presence of 1 mol % of metal chlorides were subjected to elemental analysis. Crystals were isolated by filtration and ground into powders. A Thermo Flash 2000 Organic elemental analyzer was then used to determine the atomic fraction of C, H, and N in the sample, and a Thermo Scientific iCAP 6300 (using inductively coupled plasma optical emission spectrometry (ICP-OES)) was used to determine the fraction of metal present.

### 2.6 Fluorescence Microscopy

An aqueous solution of glycine *S* = 1.02 containing 1 mol % l-Trp was prepared and held at 15 °C in a jacketed vessel. An α-gly seed crystal was then added and left to grow for 5 days, after which time it was removed and examined by fluorescence microscopy to detect any incorporation of l-Trp in the different facets of the crystal (Leica M205 FA stereo fluorescence microscope).

### 2.7 pH Measurements

The pH of growth solutions was measured at room temperature using an InLab Expert Pro-ISM electrode connected to a Mettler Toledo pH meter.

### 2.8 X-ray Photoelectron Spectroscopy (XPS)

An ESCA2SR spectrometer (ScientaOmicron GmbH) equipped with monochromated Al Kα radiation (1486.6 eV, 20 mA emission at 300 W) was used to collect XPS data to investigate the incorporation of metals onto specific crystal facets. A charge neutralizer (FS40A electron flood gun from PREVAC) was used for charge compensation, and measurements were performed under ultrahigh vacuum (UHV) conditions with pressures <10^–8^ mbar. Binding energy calibration was performed using the C–C component of C 1s (present for adventitious carbon); the peaks were calibrated to 285 eV. High-resolution scans were then performed on the following regions: C 1s, N 1s, O 1s, Cl 2p, Zn 2p, Cu 2p, and Fe 2p. Analysis of the data and curve fitting was performed using Casa XPS software. Large (>0.5 cm) crystals were mounted onto the sample holder using carbon tape so that the facet of interest was orientated parallel to the sample holder.

### 2.9 Cambridge Structural Database (CSD) Search

The CSD^36^ was searched for metal complexes of Fe, Cu, and Zn with gly allowing for either gly^–^ or Cl^–^ for counterions and H_2_O as additional ligands using Conquest.

## 3 Results

### 3.1 Brief Overview of Gly Forms and α-Gly

At present, there are six known polymorphs for gly. The first three polymorphs (α, β, and γ) are widely known and have been widely studied under numerous conditions,^16^ while the other three are obtained at high pressures (δ, ε, and ζ).^33,37,38^ While γ is the stable form at room temperature and atmospheric pressure, the metastable α form is most readily obtained by crystallization from aqueous solutions and is the subject of the current work.

**Figure 2:**
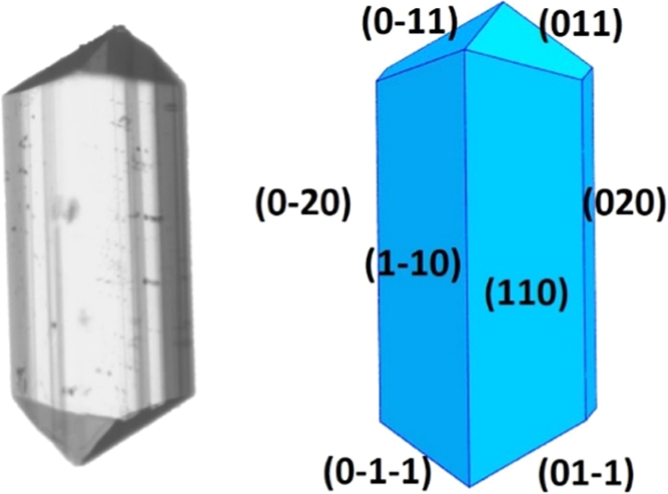
Typical morphology of an α-gly crystal grown from an aqueous solution (left) snapshot of a crystal, (right) drawing with crystal facets labeled.

**Figure 3:**
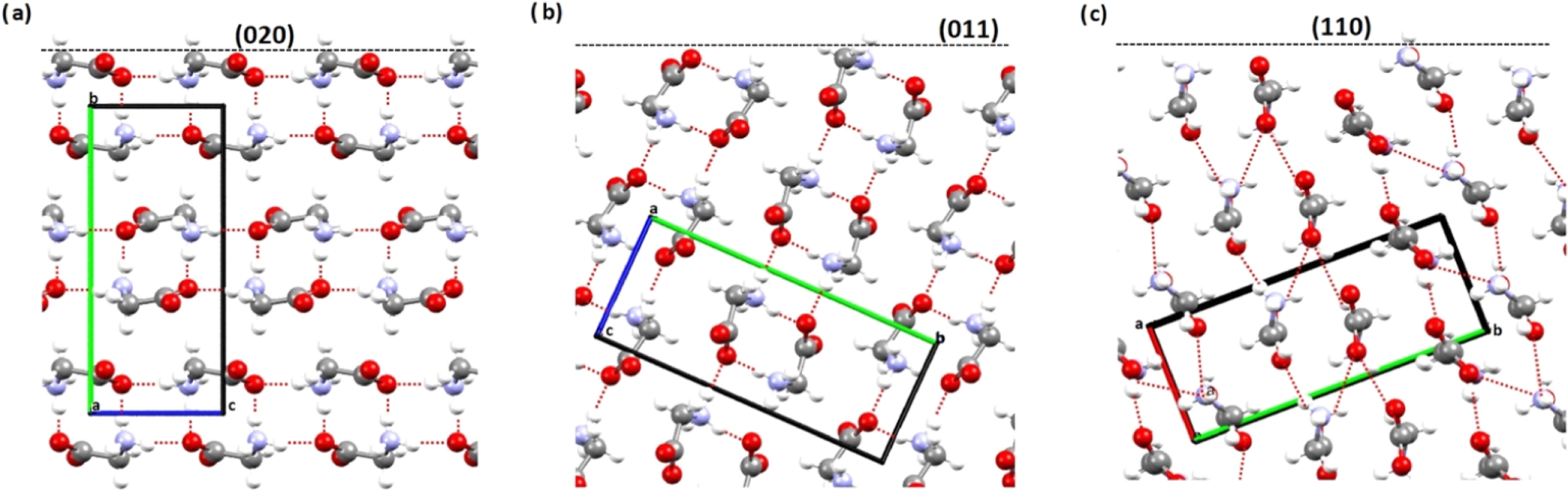
Packing view of the α-gly crystal structure showing the structures of the (020) plane (a), the (011) plane (b), and the (110) plane (c).

α-Gly (with a Cambridge Structural Database refcode GLYCIN02) crystallizes in the monoclinic *P*2_1_/*n* space group with all gly molecules as zwitterions.^39^ Briefly, the crystal structure consists of hydrogen-bonded (HB) layers containing gly dimers, which stack along the *b*-axis, making use of weak CH···O contacts. Crystals of α-gly grown from aqueous solutions have a chunky elongated morphology exhibiting three families of faces, {110}, {020}, and {011}, as shown in Figure 2, with the molecular packing of the different faces shown in Figure 3. The 2D images obtained from our setup can only gather information on the crystal facets {011} and {020}, which determine the length and width of the α-gly crystals. Typically, the {011} facets grow two to three times faster than the {020} facets in aqueous solutions, which gives α-gly its elongated shape. Growth on the (011) face involves the attachment of gly dimers through HBs, while growth on the (020) face involves the stacking of HB layers through softer CH···O interactions (Figure 3).

### 3.2 Solubility Values

The aqueous solubility values of pure α-gly as well as α-gly in the presence of the various additives at 1 mol % are given in Table 1. Our solubility of pure α-gly in water is in good agreement with values reported in the literature.^40^ α-Gly solubility remains mostly unaltered in the presence of these additives at the 1 mol % concentration studied here (Table 1). A noticeable increase in the solubility is seen in the presence of MA and CuCl_2_.

**Table 1:** Aqueous Solubility of α-Gly in the Presence of 1 mol % of Various Additives at 15 °C

additive	solubility (mol fraction)
no additive	0.0474 ± 0.0010
l-methionine	0.0469 ± 0.0003
L-tryptophan	0.0474 ± 0.0004
iminodiacetic acid	0.0478 ± 0.0001
malonic acid	0.0488 ± 0.0002
salicylic acid	0.0476 ± 0.0006
copper chloride	0.0499 ± 0.0001
iron chloride (tetrahydrate)	0.0475 ± 0.0002
zinc chloride	0.0477 ± 0.0007

### 3.3 Growth of α-Gly in Water in the Presence of Organic Additives

While α-gly is not chiral and crystallizes in a centrosymmetric space group, enantiopure chiral additives will attach preferentially to only one of the enantiotopic {020} faces of α-gly. This was first reported by Weissbuch et al.,^41^ and it was established that *R*-enantiomers of effective additives attach to the (020) face of α-gly (+*b* growth direction) while S-enantiomers of effective additives attach to the (02̅0) instead. This is relevant for our work since our l-amino acids of S handedness if they were acting as effective additives, should preferentially attach to the −*b* growth direction. None of the other facets of α-gly are enantiotopic, so their growth should be symmetrical.

**Figure 4:**
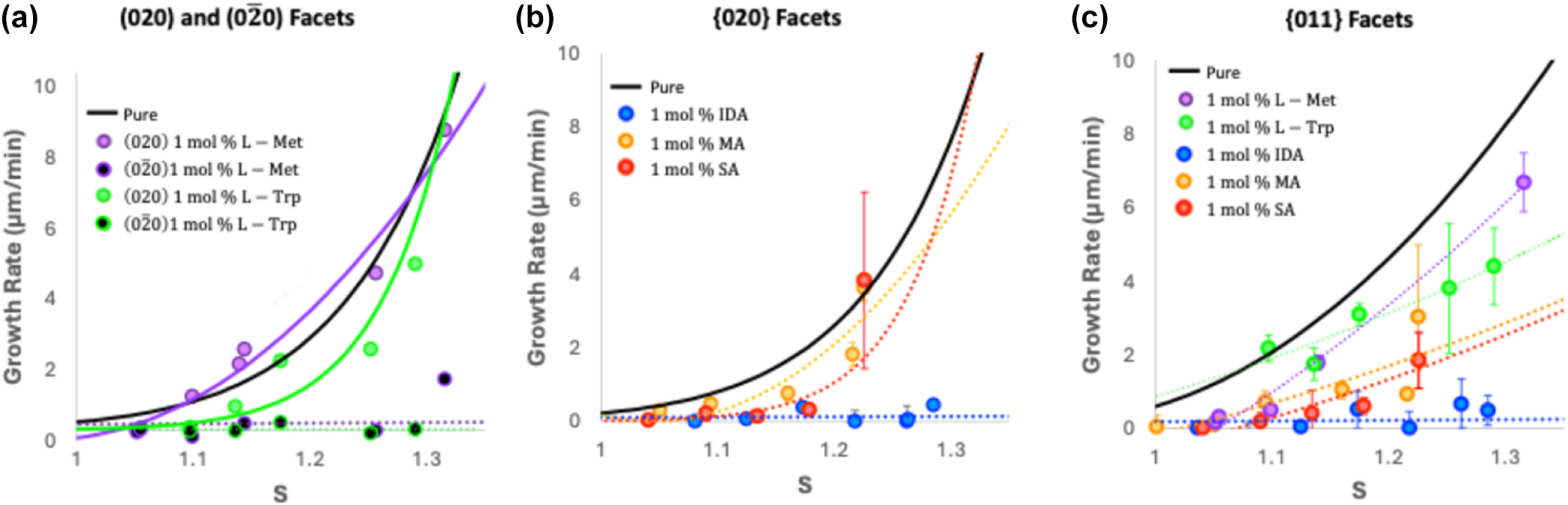
α-Gly growth rates for: (a) {020} faces grown in the presence of 1 mol % l-Met and l-Trp, (b) {020} faces grown in the presence of 1 mol % IDA, MA and SA, and (c) {011} faces for all systems in water at 15 °C. Growth rate data for pure α-gly, from our previous work, is shown as the thick black trend line.^34^ Trend lines have been added as dashed or colored solids as visual aids to highlight the additive effects.

The growth rates of α-gly crystals in the presence of organic additives are summarized in Figure 4. Figure 4a shows the impact of l-Met and l-Trp on the growth of the {020} faces, Figure 4b shows the effect of IDA, MA, and SA on the average {020} faces, and Figure 4c shows the effect of all systems on the {011} faces. For families of facets, {020} and {011}, growth rates for all symmetrically related faces are reported as an average with its standard deviation. We note that standard deviations can get larger at higher supersaturation values. This may be a consequence of multiple mechanisms of growth playing a role and also the impact of specific facet defects on those. For chiral additives interacting differently on the (020) face or (02̅0) face, only one growth rate is derived, one per unique enantiotopic face (Figure 4a).

For the chiral additives, l-Met and l-Trp, the growth along the enantiotopic {020} faces is separated since rates are significantly different, while for the other additives and nonenantiotopic faces, the average growth of both facets with the deviation is shown (error bars in Figure 4). For comparison, the growth rate of pure α-gly is also given as a black trend line with data points previously reported in ref (34).

Regarding the growth of α-gly in the presence of the two l-amino acids studied (green and purple data points Figure 4a,c), they seem to be very effective at inhibiting growth along the −*b* axis, attaching on the enantiotopic (02̅0) face of α-gly and hence slowing down its growth, while having no noticeable effect on the growth of the other facets. There is an indication that S-Trp, with its bulkier substituent, is a slightly more effective growth inhibitor than S-Met for the (02̅0) face, but the differences are small. These results are in agreement with reports on the growth of α-gly in the presence of l-alanine.^41^ Both MA and SA have a minor impact on the growth rates of the {020} faces but cause a noticeable inhibition effect on the {011} growth (Figure 4b,c). SA appears to be significantly more effective than MA at inhibiting the growth of the {011} facets (Figure 4c). Finally, IDA was found to be a very effective crystal growth inhibitor of α-gly for both the {020} and {011} family of facets across all supersaturations tested (Figure 4b,c).

We note that the pH of the solution may affect α-gly solubility and crystal growth due to the increased presence of glycine anions and cations. However, for all of the experiments studied here, the pH of the solutions with 1 mol % additives was ∼4.6 ± 0.1, which is within the pH range where solubility is essentially constant because most of the gly is found in its neutral zwitterionic form (cf. p*K*_a_ values of gly). The l-amino-acid additives, as explained in the Section 1, exist as neutral zwitterionic forms while IDA, SA, and MA exist as the anionic form mostly.

### 3.4 Growth of α-Gly in Water in the Presence of Metal Salts

**Figure 5:**
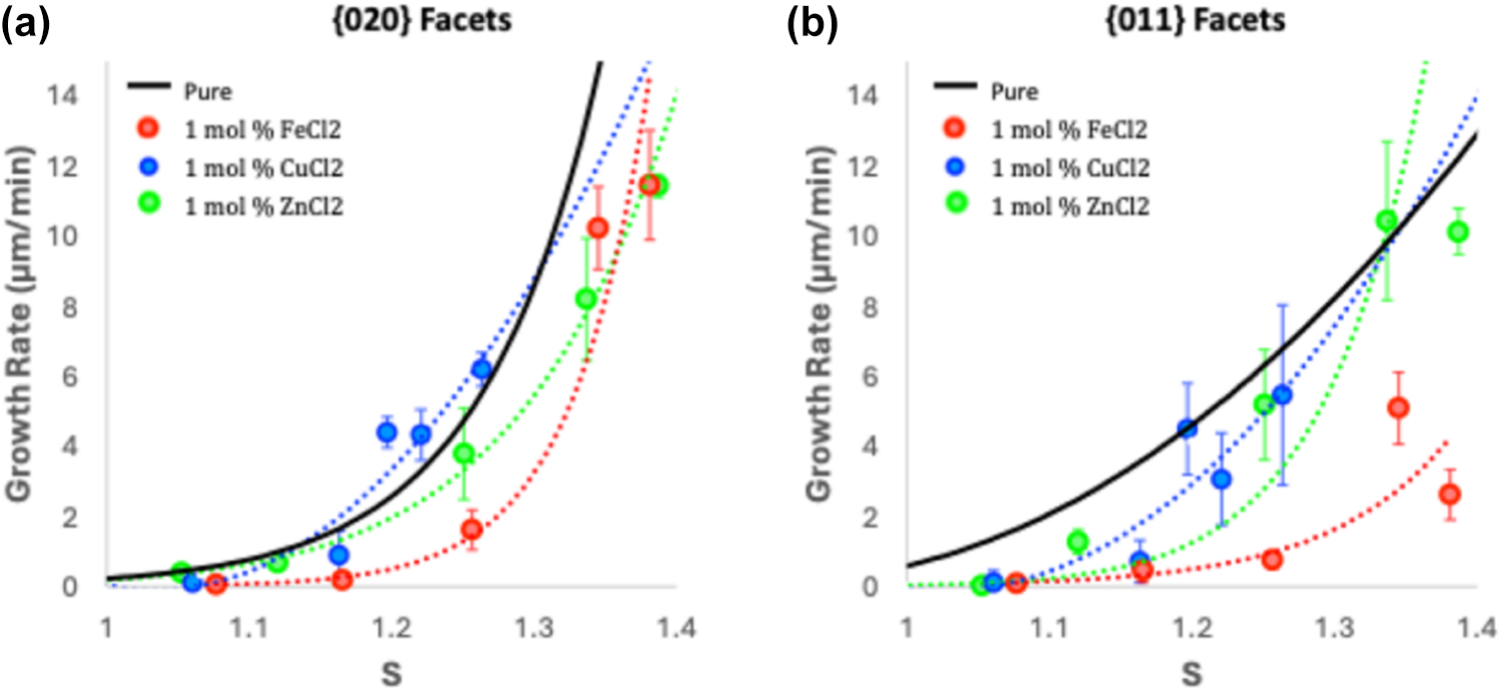
Growth of α-glycine (a) {020} and (b) {011} facets in the presence of 1 mol % FeCl_2_, CuCl_2_, and ZnCl_2_ in water at 15 °C. Growth rate data for pure α-gly, from our previous work, is shown as the thick black trend line.^34^ Trend lines have been added as dashed or colored solids as visual aids to highlight the additive effects.

The growth rates of α-gly crystals in the presence of the three metal salts studied are summarized in Figure 5. Overall, the impact on the growth rates for these additives is negligible except for FeCl_2_ on the {011} faces, as seen in Figure 5b.

### 3.5 Morphological Changes and Additive Incorporation

**Figure 6:**
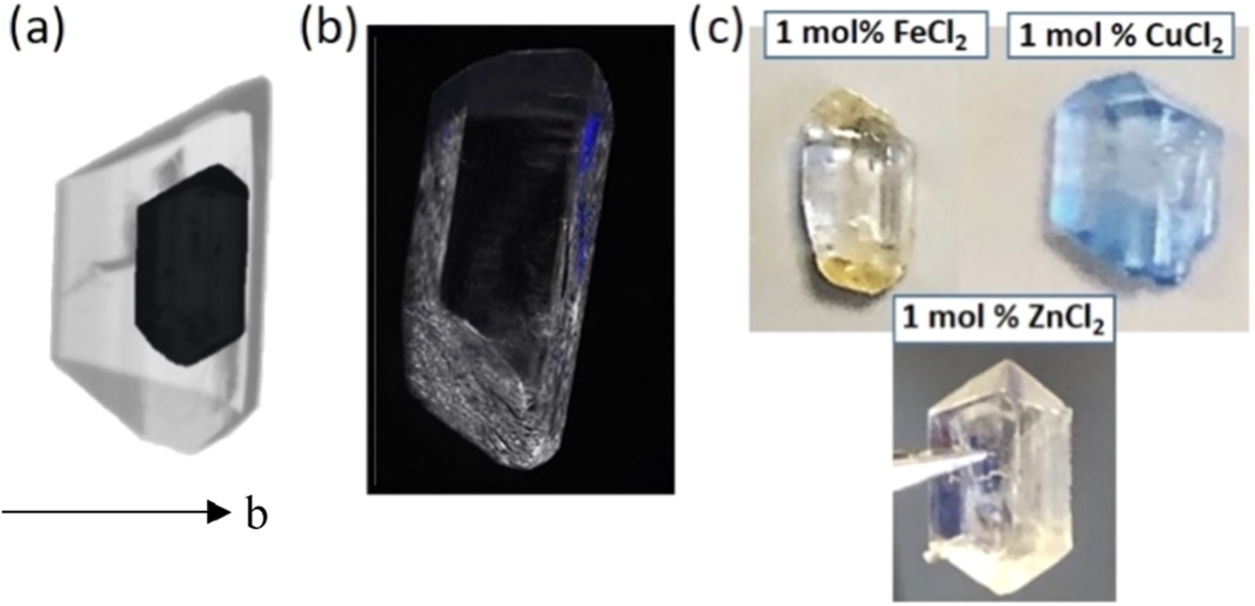
Facet specific additive incorporation into α-gly crystals. (a) l-Met adsorbs onto the (02̅0) face, dramatically impacting the symmetry of the α-gly crystal. The image overlays the pure α-gly crystal seed at the beginning of the growth experiment and the resulting crystal after 100 min of growth at *S* = 1.31 in the presence of 1 mol % of l-Met. (b) l-Trp (blue coloring) absorbed on the (02̅0) face of α-gly crystals imaged with fluorescence microscopy. (c) Metal ions adsorb on the {011} faces of α-gly crystals, grown as *S* = 1.45, as seen by the change in coloring in the crystals grown in the presence of Fe(II) and Cu(II).

In the previous sections, we have shown the impact of the various additives studied on the growth rates of the {020} and {011} facets of α-gly. In this section, we look at variations of morphologies of α-gly grown with these additives, which are dictated by the changes in growth rates, and we use fluorescent and optical microscopy to probe the specific facet incorporation of some of the additives (Figure 6).

For the l-amino acids, we have shown that the growth rate of the (02̅0) face of α-gly is impacted most. Images of the α-gly crystals grown in the presence of l-Met and l-Trp show a dramatic reduction in crystal symmetry, with the mirror symmetry perpendicular to the *b*-axis and one of the {011} facets disappearing (Figure 6a,b). A crystal of α-gly grown in the presence of 1 mol % of l-Met for 100 min is shown in Figure 6a with the original seed crystal (in black) overlaid. Since l-Trp is fluorescent, imaging of an α-gly crystal grown in the presence of l-Trp reveals blue fluorescing regions selectively located on the (02̅0) face of the crystal only. This reconfirms, again, the selective uptake of the L-amino acids on the (02̅0) face of α-gly.

For the organic acids, morphological changes are much less noticeable (not shown). With SA, the crystals become less elongated; with MA, the changes are small; and with IDA, there are no changes since the growth is significantly inhibited in all facets.

In the case of the metal ions, morphological variations are less dramatic, with the crystal shape of α-gly remaining similar to the seed used during growth (Figure 6c). Thanks to the color of the Cu(II) and Fe(II) ions, however, simple optical imaging reveals the selectivity in the incorporation of these ions. Figure 6c clearly shows that the Fe(II) ions incorporate selectively into the {011} faces, resulting in the visibly yellow coloration of the {011} facets. Similarly, Cu(II) ions turn α-gly {011} facets dark blue, with lighter blue coloration also perceived in the rest of the crystal. Finally, for ZnCl_2_, while no coloration was observed, similarly to Cu(II) and Fe(II), Zn(II) is known to form complexes with gly, so we expect it could also incorporate into the crystal through the {011} with a similar mechanism.

### 3.6 Quantification of Additive Incorporation

NMR, elemental analysis, and XPS techniques were used to quantify the amount of additive incorporated into the growing α-gly crystals, with XPS being used for surface quantification specifically. In the case of the organic additives, incorporation of the additives into the crystal lattice (forming solid solutions) is not expected since they are significantly larger molecules than glycine. Hence, only surface adsorption is expected. For these organic additives, solution NMR did not reveal any incorporation into the α-gly crystals. In the case of l-Met, which contains a heavier S atom, XPS was also used to probe the adsorption of the additive on the (02̅0) faces of α-gly crystals, but the mol % of additive adsorbed was not enough for detection. Elemental analysis and XPS, however, allowed for successful quantification of metal incorporation into the α-gly crystals, with the obtained data summarized in Table 2. Elemental analysis allows for quantification of the metal incorporated in the entire bulk with XPS for quantification on specific surfaces. For XPS characterization, relatively large (>0.5 cm) α-gly crystals were grown in order to be able to orient the crystal in the desired way for analysis of the different facets. In performing the XPS measurements, difficulties were encountered in mounting the crystals and positioning the X-ray beam such that only the desired {011} or {020} facet was sampled by the beam. This, together with variations in the concentration of metal adsorbed in the various samples and crystal surfaces, means that the results summarized in Table 2 should be interpreted in terms of the trends rather than absolute values.

**Table 2:** Mol % of Metal Incorporated into the Bulk (Measured with Elemental Analysis) and Surfaces (Measured with XPS) of α-Gly Crystals Grown in the Presence of 1 mol % of Metal Salt from Aqueous Solutions at *S* between 1.3 and 1.45

metal ion	mol % of metal in bulk (%)	mol % of metal in {011} (%)	mol % of metal in {020} (%)
Fe(II)	0.27	12	0^a^
Cu(II)	0.14	5	0^a^
Zn(II)	<0.1^a^	2	0^a^

a Below detection limit.

Both elemental analysis and XPS data for all metals are given in Table 2, with both techniques showing metal efficiency incorporation: Fe(II) > Cu(II) > Zn(II). No metals are detected in the {020} facets, but a significant amount of metal is incorporated on the {011} faces: from 2 mol % for Zn(II) to 12 mol % for Fe(II), with Cu(II) incorporating at 5 mol %. These data align well with the growth rate data, where Fe(II) causes a more significant inhibition of the {011} growth rate than the other two metals, presumably because of its higher efficiency in incorporating into the α-gly crystals.

## 4 Discussion

In this section, the observed effects of the additives on crystal growth are interpreted in terms of their potential interaction at the α-gly crystal surfaces.

**Figure 7:**
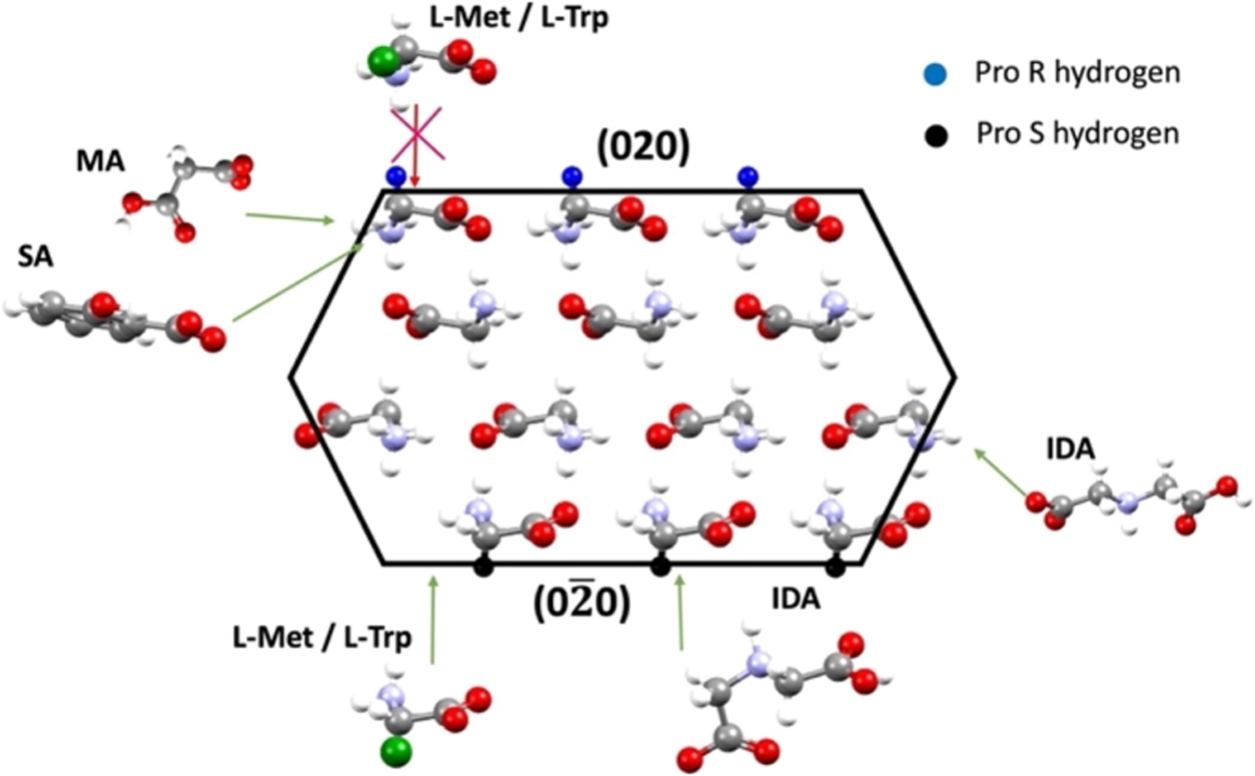
Interaction of the organic additives at the α-gly crystal surface.

For the impact of the l-amino acids, the reasoning follows the original work of Weissbuch et al.,^41^ who showed that the impact of R and S amino acids on the morphology of glycine originated in the enantiotopic (prochiral) nature of the {020} faces with (R) additives affecting the growth and being incorporated stereoselectively in the (020) faces and (S) additives in the (02̅0) faces. Accordingly, Figure 7 shows the arrangement of gly zwitterions at the {020} and {011} surfaces of α-gly. The hydrogens exposed at the {020} surfaces are prochiral, a feature emphasized in Figure 7 with the pro-(R) hydrogen atoms exposed at the (020) surface colored blue and the pro-(S) hydrogen atoms exposed at the (02̅0) surface colored black. The l-amino acids with (S) handedness, such as l-alanine,^41^l-leucine,^42^l-aspartic acid,^43^ and l-glutamic acid,^43^ have been shown to attach to the (02̅0) faces preferentially through this mechanism, and we have shown here the same effect for l-Met and l-Trp from our growth rate measurements and fluorescence microscopy. Figure 7 shows how this selectivity is consistent with the stereochemistry of the l-amino acid additives, their bulky substituents (depicted in green) preventing incorporation at the (020) face while allowing it on the (02̅0). Once incorporated into the (02̅0) face, the large groups block additional gly molecules from joining the surrounding structure and slow the growth, enhancing the morphological importance of the face. At the {011} surface, the amino acids cannot join the structure due to steric interactions caused by the R groups. The kinetic data (Figure 4) suggests that the size of the R group on the amino acids may have some effect on its inhibiting power with growth in the presence of the smaller S-Met taking off at *S* > 1.3.

For the acids, the inhibition of growth is on the {011} faces, with SA having a more significant inhibition power than MA. In this regard, the size of the acid may again play a role, with the larger molecule being more different from gly having a greater inhibiting power. These results agree with the previous literature, where malonic acid^29^ and ethylene diamine^26^ were shown to slow the growth of the {011} facets. In fact, malonic acid has not only been shown to increase the morphological importance of the {011} face in α-gly but also gave γ-glycine when doing crystallizations at higher acid content.

Growth rate data for IDA suggest that IDA can incorporate into both the {020} and the {011} facets. Under the experimental conditions, both IDA and gly will be present as zwitterions in solution. MA and IDA are almost identical in chemical structure with one difference: the central C atom in MA is replaced by an N atom in IDA. It is this amine functionality and the consequent existence of IDA in the zwitterionic state that make it an effective inhibitor of both facets, while MA and SA can only bind to the structure via the {011} facet. This is shown diagrammatically in Figure 7. Due to its ability to suppress the growth of the {020} and {011} facets, it was considered that IDA may promote the crystallization of γ-gly rather than the α-gly. Experimental results (Supporting Information (SI)) do indeed support this possibility when glycine is grown by slow evaporation and slow cooling from aqueous solutions.

**Figure 8:**
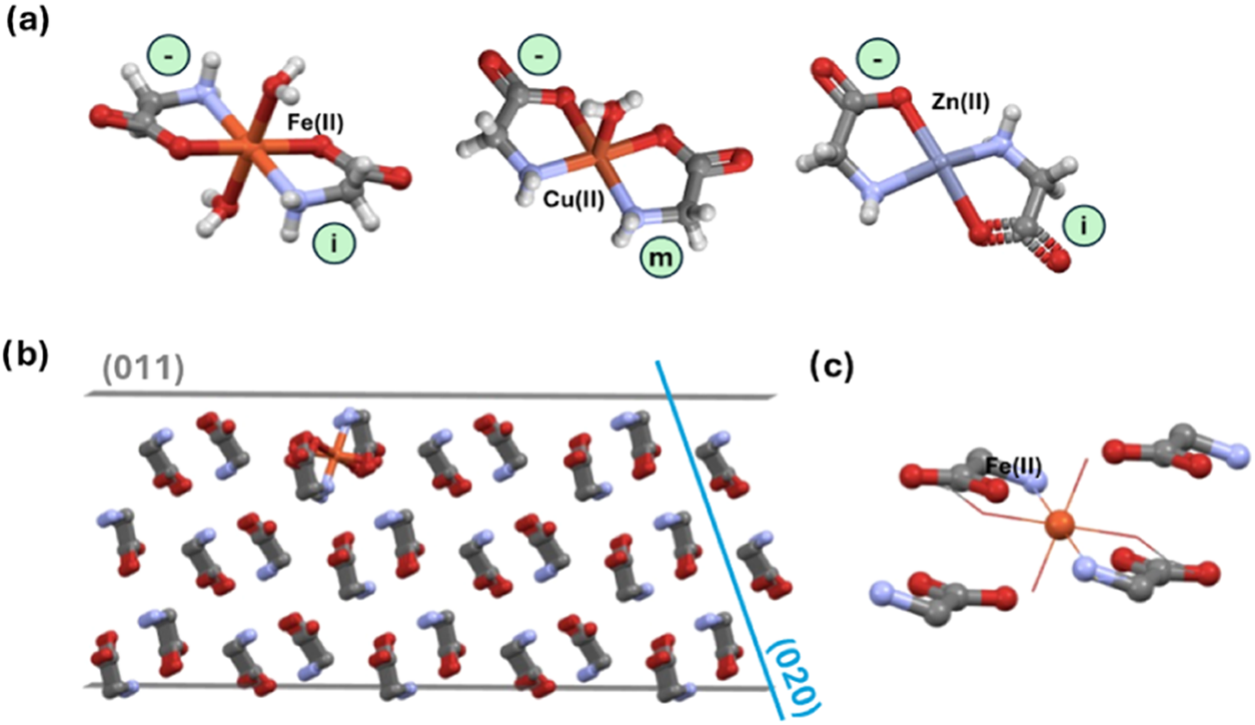
(a) Complexes formed between Fe(II), Cu(II), and Zn(II) with glycinate ligands. The symmetry relationship between glycinate molecules is indicated, with m being mirror symmetry and i being inversion. The Zn(II) complex is further coordinated with a neighboring glycinate complex, forming a catena. This bond has been removed for the purpose of the illustration. (b) Fe(II) complex incorporated into the (011) face of α-gly. (c) Overlay of the Fe(II) complex and four molecules of glycine in α-gly.

For the metal salts, we have shown that the metal ions show relatively high incorporation into α-gly crystals, but they however have relatively small effects on the morphology or the growth rates. Incorporation of the metals with the Cl^–^ is not possible since XPS confirmed that Cl^–^ are not present in α-gly grown in the presence of metal chlorides. The metals must thus form coordination complexes with the glycinate ligands. This theory is supported by the fact that XPS showed that Fe(II) electrons are in a high-spin arrangement—which would be expected for an iron bisglycinate complex.^23^ A search of the CSD showed that all three metal ions (Fe(II), Cu(II), and Zn(II)) are able to form planar complexes with two bidentate glycinate molecules (Figure 8a). The metals then further coordinate to either water molecules (as for Fe(II) and Cu(II) with coordination of 6 and 5, respectively), or neighboring glycinate molecules catenating into a polymer—for Zn(II). Figure 8 shows the symmetry of the glycinate molecules around the metal ions, with the Fe(II) complex having the two glycinate molecules related by inversion, the Cu(II) by mirror symmetry, and the Zn(II) by inversion. The distance between the glycinate molecules in the complex is very similar to the gly dimers in α-gly, therefore having a perfect size match for their incorporation. Figure 8b shows the iron complex incorporated into the α-gly (011) face, illustrating the perfect fit of the Fe(II) glycinate complex into the α-gly lattice, with the coordination of the two water molecules being easily replaced by gly molecules in the structure (Figure 8c).

Regarding the effectiveness of the metal incorporation into the lattice, we note that the Fe(II) bisglycinate complex known in the CSD has the correct glycinate symmetries (matching the gly dimers related by inversion) and the correct coordination number required for the 6 coordination pocket within the α-gly lattice (Figure 8c). The known Cu(II) glycinate complex, however, has the glycinate molecules related by mirror symmetry and a coordination number of 5. Finally, while the Zn(II) has the correct symmetry for the α-gly lattice, it is known to form catenate structures with glycinate. If those catemeric structures are forming in solution, then it is expected that those will not be able to incorporate into the α-gly lattice. These rationalizations match well the XPS data, with the Fe(II) metal having the best incorporation into α-gly, with both Cu(II) and Zn(II) having significantly less.

Regarding the facet selectivity for {011} incorporation of the glycinate metal ion complexes, the best explanation may lie in the fact that those billycinate complexes are preformed in solution and incorporated as such in the growing crystal. Work by Yani et al.^200^ has shown that gly dimers of similar geometries to the glycinate complexes incorporate more favorably on the {011} than the {020} facets through significantly stronger interaction energies. This energetic preference for the dimer attachment on the {011} provides an explanation for the observed facet selectivity.

## 5 Conclusions

Many works on assessing the impact of additives on crystal growth have been inferred from morphological observations^41^—growth inhibition leading to increased morphological importance of the face affected. This work extends previous studies^26,42−45^ and shows how kinetic data can support this understanding and allow rapid assessment of additive effects (including stereochemical). It also ensures that the effect of inhibitors that affect the growth of all facets to a similar extent is not missed.

Here, we have studied the impact of a range of additives of different natures on the growth kinetics of α-gly. We have used a recently developed setup that allows for the study of crystal growth under flow conditions and produces growth rates as a function of supersaturation and additive content efficiently from images of a single crystal exposed to cycles of dissolution and growth. Since the growth and dissolution cycles are automated, the collection of data takes a third of the time compared to a manual system.

Our new data on α-gly growth rates allowed us to reconfirm the effect of chiral amino acids, where growth is affected asymmetrically due to the prochirality of α-gly.^41^ The inhibiting effect on rates of S-Trp is slightly more effective than S-Met, which could be rationalized because of the larger size of S-Trp. However, this difference is small. In the case of MA and SA, the importance of species charge on its inhibiting effect is seen. Also, when comparing MA and SA to IDA, the consequence of structural and chemical similarity between the solute and additive is highlighted, in this case, leading to a highly effective growth inhibitor. We also observe the incorporation of metal ions and their unexpectedly limited impact on the growth rate. We relate this observation to the ability of the metal ion to form complexes with glycinate, akin to the cyclic dimers present in the α-gly. Further to the kinetics data on rates, we also use analytical techniques to quantify the additive incorporation on the different faces, providing further experimental quantitative evidence on the effect of such additives on the different facets. Understanding crystal growth and additive effects is key knowledge that can help us develop improved crystallization processes at larger scales.

## Supporting Information Available

XPS sample mounting (Figure S1) and data information (Figures S2–S4), data on crystallization outcomes of glycine in the presence of IMDA, CSD analysis of glycine structures containing metals (Cu, Zn, and Fe), and data on crystallization outcomes of glycine in the presence of metals (PDF)
