# Supplementary material for: Investigating Additive Effects on α-Glycine Growth through the Measurement of Facet Specific Growth Rates
Source: Cryst Growth Des. 2025 Feb 13;25(5):1644–52. doi: 10.1021/acs.cgd.5c00028 (PMC11887511; doi:10.1021/acs.cgd.5c00028)
Supplement: Supplementary file 1 — cg5c00028_si_001.pdf [file cg5c00028_si_001.pdf]

# Supporting Information for:

## Investigating Additive effects on α-Glycine Growth through the Measurement of Facet Specific Growth Rates

Caroline Offiler,[a] Roger J. Davey,[a] and Aurora J. Cruz-Cabeza*[a,b] and Thomas Vetter*[c]

[a]Department of Chemical Engineering, University of Manchester, Manchester M13 9PL, United Kingdom.

[b]Department of Chemistry, Durham University, Durham DH1 3LE, United Kingdom.

[c]H. Lundbeck A/S, Valby 2500, Denmark.

*Corresponding authors emails: THVT@lundbeck.com and aurora.j.cruz-cabeza@durham.ac.uk

## 1. XPS Data

Figure 1 shows how α-gly crystals were mounted on to the samples holder so that a {011} facet could be analysed by XPS.

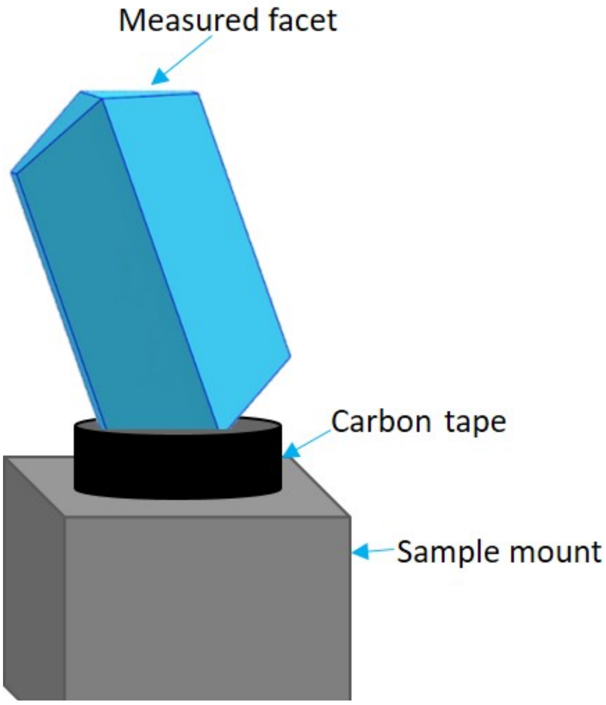


**Figure 1.** Positioning of a crystals on the sample mount for analysis of a {011} facet.

XPS spectra is shown in Figures 2-4. We also note that not all glycine is in the zwitterionic form this may occur form surface contamination or interactions with contaminates.

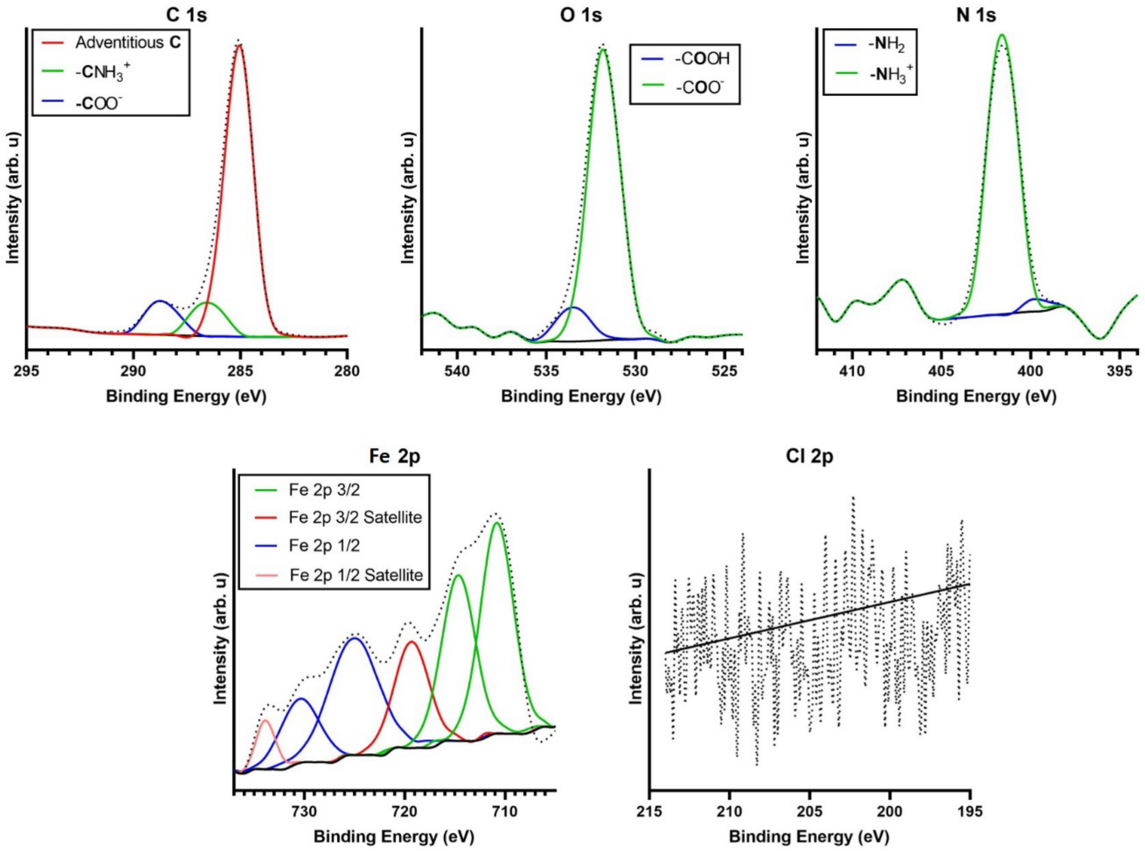


**Figure 2.** XPS spectra taken from a {011} facet of a α-gly crystal grown in the presence of 1 mol% $FeCl_2$.

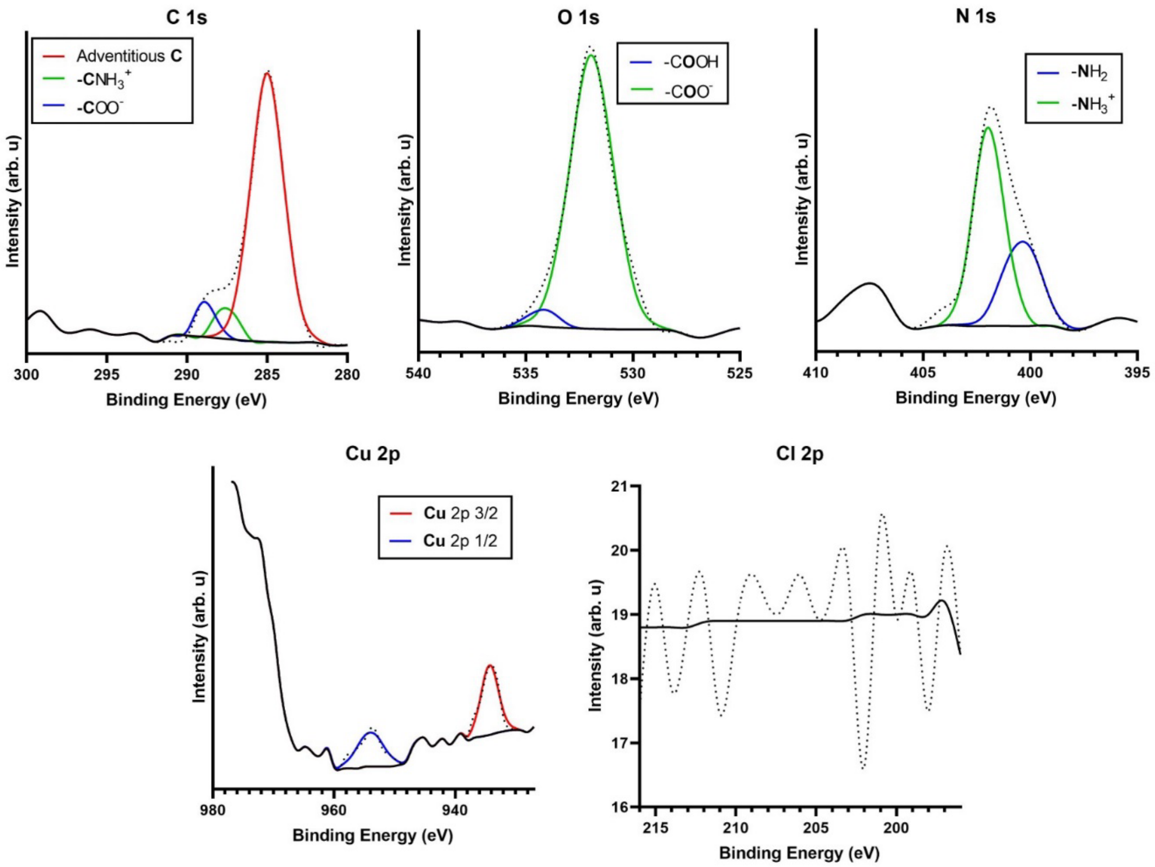


**Figure 3.** XPS spectra taken from a {011} facet of a α-gly crystal grown in the presence of 1 mol% $CuCl_2$.

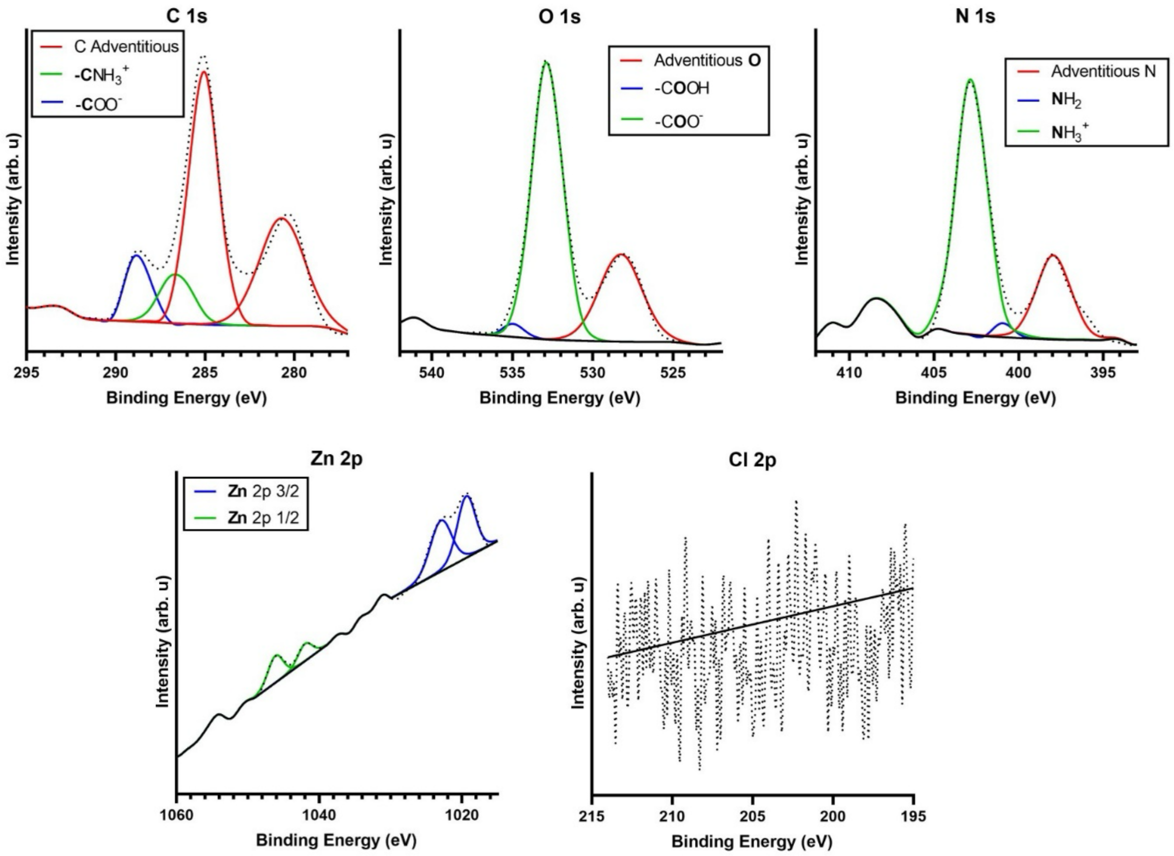


**Figure 4.** XPS spectra taken from a {011} facet of a α-gly crystal grown in the presence of 1 mol% $ZnCl_2$.

## 2. IDA on Glycine Polymorph

To test if IMDA would promote the nucleation of γ-gly over α-gly both slow cooling and slow evaporation experiments were performed.

For slow evaporation experiments the appropriate amount of gly, IMDA and water were added to a glass vial which was stirred at 30°C for approximately until all the solid had dissolved. The stirrer bar was removed and the vales were covered with pierce parafilm and left until crystals were seen.

For slow cooling experiments solutions of gly in water were made (0.251 g gly per g water) to each solution IMDA was added (1-5 mol% compared to gly). Solutions were stirred at 30 °C for approximately 2 hrs until all the solute has dissolved after which time the stirrer bar was removed and solutions were cooled to 15 °C at 1 °C per hr.  Solutions were held at 15 °C until crystals were seen.

In both cases crystals were isolated by vacuum filtration crushed into a powder then analysed by PXRD.

Results are shown in Table 1, in the absence of IDA we would expect all samples to show the α form only so it would appear that in some cases IDA inhibits α-gly growth allowing γ-gly growth. However the results are inconsistent and such it does not appear that IDA could be used to selectively crystallise γ-gly.

**Table 1.** Results of experiments to determine the effect if IDA on glycine polymorph.

| Method | mol% IMDA (w.r.t. gly) | Phases in PXRD |
|---|---|---|
| Slow evaporation | 0.181 | α + γ (trace) |
| | 0.505 | α |
| | 0.798 | α |
| | 1.135 | α + γ |
| | 1.572 | α |
| | 5.510 | γ |
| | 10.364 | α |
| Slow cooling | 1.011 | α |
| | 1.986 | α |
| | 5.012 | α + γ (trace) |
| | 5.047 | α |

## 3. Metal Glycine Complexes

Table 2 shows the metal glycine/glycinate complexes found by a CSD search. The geometry of the complex and the denticity of the glycine/glycine ligands is also given. Figure 5 shows the metal biglycinate complexes alongside the dimer 2 structure present in the α-gly structure.

**Table 2.** Know complexes metal (Cu, Zn or Fe) glycine complexes with only $H_2O$ or $Cl^-$ as additional ligands.

| Complex | Coordination Geometry | Ref Code | Glycine Denticity |
|---|---|---|---|
| $[Fe(Gly^-)_2(H_2O)_2]$ | Octahedral | OFANER | Bidentate |
| $[Cu(Gly^-)_2H_2O]$ | Square pyramidal | CUGLYM | Bidentate |
| $[Zn(Gly^-)_2].H_2O$ | Planer | ZZZDVY | Bidentate |
| $[Zn(Gly\pm)Cl_2H_2O]$ | Tetrahedral | NOHBIW | Monodentate |
| $[Zn(Gly\pm)_2Cl_2].H_2O$ | Tetrahedral | AFEMEE | Monodentate |
| $[Zn(Gly\pm)_2Cl_2].(Gly\pm)$ | Tetrahedral | KEDDIG | Monodentate |

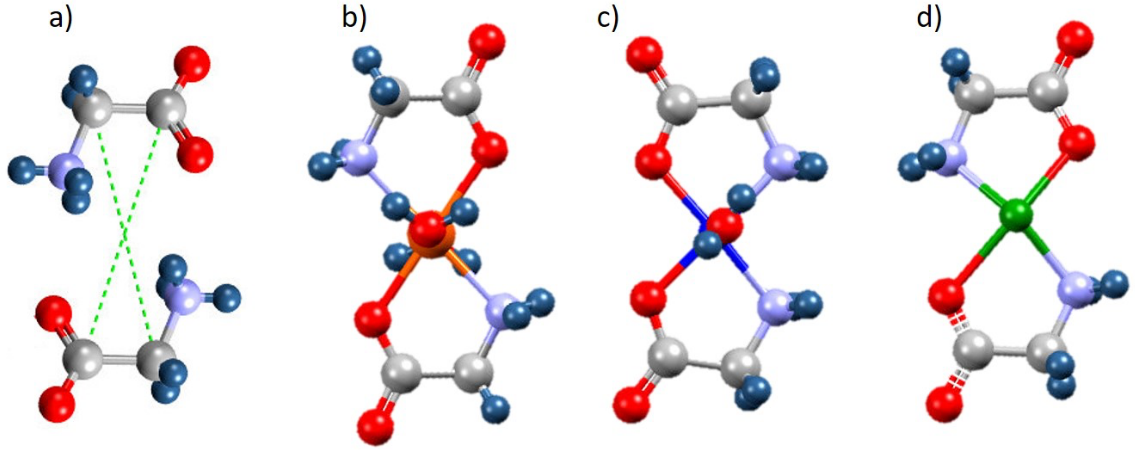


**Figure 5.** a) glycine dimers present in the α-glycine crystal strucuter alongside complexes fromed between metals ions and glycinate ligands b) Fe(II), c) Cu(II) and d) Zn(II).

## 4. Tests with $CoCl_2$

To examine the coordination environment of metal complexes incorporating into the crystal structure slow cooling experiments were performed in the presence of 1 mol% $CoCl_2$.

Two aqueous solutions were prepared containing gly and 1 mol% $CoCl_2$. Solutions were stirred at 30 °C for approximately 2 hrs until all the solute has dissolved after which time the stirrer bar was removed and a α-gly seed crystal was added to one of the solutions. The solutions were cooled to 15 °C at 1 °C per hr (approximate final supersaturation is 1.3). Solutions were held at 15 °C until crystals were seen these were isolated by vacuum filtration.

The resulting crystals are shown in Figure 6, however incorporation is less clear to see visually than with Fe(II) and Cu(II). The seeded crystal (Figure 6 a) shows faint pink colouring is seen on the {011} facets the crystals in Figure 6b show regions of pink colour (the contract of the image has been enhanced to show this. The pink colour indicates Co(II) in a octahedral environment which may be the result of a complex formed with gly in solution or solvent inclusion. A search of the CSD data base revels no Co(II) bidentate glycinate complexes similar to those found for Fe(II), Cu(II and Zn(II) which may explain its lower incorporation.

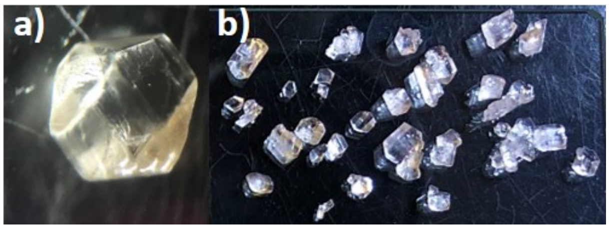


**Figure 6.** Glycine crystals grown by slow cooling in the presence of 1 mol % $CoCl_2$. a) Seeded and b) unseeded.
